# Exploration of a machine learning approach for diagnosing sarcopenia among Chinese community-dwelling older adults using sEMG-based data

**DOI:** 10.1186/s12984-024-01369-y

**Published:** 2024-05-09

**Authors:** Na Li, Jiarui Ou, Haoru He, Jiayuan He, Le Zhang, Zhengchun Peng, Junwen Zhong, Ning Jiang

**Affiliations:** 1 The National Clinical Research Center for Geriatrics, West China Hospital of Sichuan University, Chengdu, Sichuan 610041 China; 2 Medical Equipment Innovation Research Center, West China Hospital of Sichuan University, Chengdu, Sichuan 610041 China; 3 The Med-X Center for Manufacturing, Sichuan University, Chengdu, Sichuan 610041 China; 4 College of Computer Science, Sichuan University, Chengdu, 610065 China; 5 School of Electronic Information and ElectricaEngineering, Shanghaijiao Tong University, Shanghai, 200240 China; 6 Department of Electromechanical Engineering and Centre for Artificial Intelligence and Robotics, University of Macau, Macau, SAR 999078 China

**Keywords:** Sarcopenia, EMG, Machine learning, SHAP, Early diagnosis

## Abstract

**Background:**

In the practical application of sarcopenia screening, there is a need for faster, time-saving, and community-friendly detection methods. The primary purpose of this study was to perform sarcopenia screening in community-dwelling older adults and investigate whether surface electromyogram (sEMG) from hand grip could potentially be used to detect sarcopenia using machine learning (ML) methods with reasonable features extracted from sEMG signals. The secondary aim was to provide the interpretability of the obtained ML models using a novel feature importance estimation method.

**Methods:**

A total of 158 community-dwelling older residents (≥ 60 years old) were recruited. After screening through the diagnostic criteria of the Asian Working Group for Sarcopenia in 2019 (AWGS 2019) and data quality check, participants were assigned to the healthy group (*n* = 45) and the sarcopenic group (*n* = 48). sEMG signals from six forearm muscles were recorded during the hand grip task at 20% maximal voluntary contraction (MVC) and 50% MVC. After filtering recorded signals, nine representative features were extracted, including six time-domain features plus three time-frequency domain features. Then, a voting classifier ensembled by a support vector machine (SVM), a random forest (RF), and a gradient boosting machine (GBM) was implemented to classify healthy versus sarcopenic participants. Finally, the SHapley Additive exPlanations (SHAP) method was utilized to investigate feature importance during classification.

**Results:**

Seven out of the nine features exhibited statistically significant differences between healthy and sarcopenic participants in both 20% and 50% MVC tests. Using these features, the voting classifier achieved 80% sensitivity and 73% accuracy through a five-fold cross-validation. Such performance was better than each of the SVM, RF, and GBM models alone. Lastly, SHAP results revealed that the wavelength (WL) and the kurtosis of continuous wavelet transform coefficients (CWT_kurtosis) had the highest feature impact scores.

**Conclusion:**

This study proposed a method for community-based sarcopenia screening using sEMG signals of forearm muscles. Using a voting classifier with nine representative features, the accuracy exceeds 70% and the sensitivity exceeds 75%, indicating moderate classification performance. Interpretable results obtained from the SHAP model suggest that motor unit (MU) activation mode may be a key factor affecting sarcopenia.

## Introduction

With the advancements in science, healthcare technology, and socioeconomic development, people worldwide are experiencing increased lifespans [1]. In 2019, approximately one billion individuals globally were over the age of 65, and it is projected to reach 1.4 billion by 2030 and 2.1 billion by 2050 [2]. Unfortunately, older individuals are often assumed to be frail or dependent and a burden on society. The increase in the elderly population has generated a growing demand for healthcare services, leading to difficulties in allocating medical resources [3]. Consequently, there has been a significant emphasis on understanding age-related chronic diseases and developing innovative approaches to tackle these challenges, thereby alleviating the strain on healthcare systems. One of the age-related musculoskeletal diseases is sarcopenia, which is characterized by a progressive and generalized loss of skeletal muscle, resulting in accelerated loss of muscle mass and physical function [4]. It has been demonstrated by researchers that sarcopenia leads to serious healthcare issues since it is associated with increased adverse outcomes including falls, functional decline, frailty, and mortality [5]. Current research indicates that the global prevalence of sarcopenia ranges from 10 to 16% [6] and even up to 29% in some communities [7]. Among individuals aged 80 years and above, this rate can be up to 50% [8].

According to the revised consensus published by the Asian Working Group for Sarcopenia in 2019 (AWGS 2019) and the European Working Group on Sarcopenia in Older People (EWGSOP), the diagnosis of sarcopenia requires measurements of a combination of muscle mass, muscle strength, and physical performance [5, 9]. Currently, there are four commonly used techniques for estimating muscle mass: bioelectric impedance (BIA), dual-energy X-ray absorptiometry (DXA), computed tomography (CT), and magnetic resonance imaging (MRI) [10–12]. However, DXA and BIA are more widely used [4], and DXA is considered the gold standard for measuring lean body mass [13]. The need for professional operation, high cost, and lack of portability with DXA restricts its practical application in community settings. On the other hand, due to its low cost and ease of use, BIA is the most widely used technique in scientific research and clinical practice, as well as being a portable tool that can be used in various settings, including community settings [14]. Nevertheless, when estimating muscle mass using BIA, there is a significant individual prediction error [15, 16]. Different from AWGS, the EWGSOP uses low muscle strength as the primary diagnostic criterion for sarcopenia [5]. It is recommended to use a handgrip test to evaluate skeletal muscle strength in individuals with sarcopenia [9]. The shift in focus from low muscle mass to low muscle strength sarcopenia diagnosis is supported by evidence including that low grip strength is associated with repeated falls [17], low grip strength is shown to be a stronger predictor of cardiovascular mortality than blood pressure [18], and that both low grip strength and leg extensor strength were associated with impaired mobility [19]. However, the accuracy of the grip strength test results can be easily influenced by factors such as the devices used and measurement protocols [9]. In conclusion, the current diagnosis process of sarcopenia is cumbersome, time-consuming, and susceptible to various factors that impede its wider applications, especially in community settings. Additionally, it is not feasible to dynamically monitor and predict muscle function in real-time, limiting its potential in early screening and timely diagnosis of sarcopenia.

Sarcopenia is associated with a decrease in muscle fiber number along with a reduction in the size, which particularly affects type-II fibers [20–22], and is accompanied by intramuscular and intermuscular fat infiltration [23, 24]. Denervation significantly contributes not only to the loss of the motor unit (MU) but also to the loss of muscle fibers, and with the decrease in the number of MUs found in aged muscle, there is an increase in the size of the remaining MUs, which is an accepted mechanism of sarcopenia [25–27]. Activated by efferent neural drive, a motoneuron generates a series of MU action potentials (MUAPs), which propagate down to the neuromuscular junction (NMJ) and then transmit to the muscle fibers. The number of MUs [28], the function of NMJ propagation [29], and the innervation of muscle fibers [30] are all crucial factors that influence the formation of APs, the special and temporal summation of which are also referred to as electromyography (EMG) signals. It has been observed that during aging, there is preferential denervation of fast fibers with reinnervation via axonal sprouting from slow motor neurons, resulting in a conversion from type II (fast) fibers to type I (slow) fibers [31], and further changes in electrophysiological properties [32]. A recent study investigated the MU firing pattern in pre-sarcopenic senior individuals with low skeletal muscle mass but normal physical functions. The results showed that MUs of the pre-sarcopenic individuals exhibited an abnormal neural input pattern yet otherwise normal hierarchical pattern [33], which is consistent with the size principle proposed by Hennenman et al. [34]. Hu et al. [35] reported that there was no difference in MU number and mean firing rate of lower extremity muscles among individuals with risk-sarcopenia, healthy elderly, and healthy young participants, but the slope of mean MU firing rate was significantly higher in the risk-sarcopenia group compared to the young group. MU properties related to MUAP shape variability, such as jitter and jiggle, have been used to evaluate the stability of NMJ propagation [36]. Gilmore et al. [37] demonstrated that MU number estimates in the lower limb muscles were similar between pre-sarcopenic and sarcopenic subjects. However, the level of near fiber jitter and jiggle was higher in sarcopenic subjects compared to pre-sarcopenic subjects. Overall, EMG could theoretically be used to identify electrophysiological abnormalities associated with sarcopenia.

Following this approach, if there is discriminative information in EMG signals between sarcopenic and healthy muscle, machine learning (ML) methods can be applied for sarcopenia screening, even detection [38]. However, to the best of our knowledge, there is limited research on this topic. And our literature search didn’t find an sEMG sarcopenia study with upper-extremity muscles, as of March 2024. Most related EMG research suffers from small sample sizes [38]. Typically, only less than 50 participants were investigated in previous sarcopenia detection research [39], with an obvious imbalance between the groups, which would lead to poor reliability of the algorithm [38–41]. Hence, for generalization purposes, a sufficiently large sample size is necessary. In addition, ML methods used in current studies are “black-boxes”, with poor or even no interpretability, which further limits their clinical value.

Thus, to provide better reliability and interpretability of EMG-based sarcopenia screening algorithms, the primary purpose of this study was to perform sarcopenia screening in community-dwelling older adults with a size of more than 100 participants and investigate whether surface EMG from hand grip could potentially be used to detect sarcopenia in the elders using ML methods with reasonable features extracted from sEMG signals during the community hand grip trials. The secondary aim is to provide interpretability of the obtained ML models using a novel feature importance estimation method.

## Methods

### Participants

Community-dwelling older residents (≥ 60 years old) were recruited continuously in four different communities in Chengdu city through recruitment advertisements, from February 2023 to August 2023. The four communities were socioeconomically diverse, providing educational, economic, and social inclusivity of the participant pool. Briefly, inclusion criteria included being able to walk without any walking aid; and no treatment for sarcopenia before the study. Exclusion criteria were as follows: (1) A self-reported history of cancer; (2) Uncontrolled or unstable diabetes, (3) Uncontrolled or unstable high blood pressure (> 150/90 mmHg); (4) Chronic organ failure in the last 5 years; (5) Cognitive disabilities; (6) Suffering from severe osteoarthritis. Finally, a total of 158 participants (Table 1) were included and completed all the experimental procedures. After being informed of the experimental procedures and associated risks, all participants provided written informed consent, and the study was conducted in accordance with the declaration of Helsinki and approved by the ethics subcommittee of West China Hospital of Sichuan University (WCHSCU_2023_317).

**Table 1:** Descriptive statistics of all participants represented as mean (±SD)

Total (*n*=158)		Male (*n*=49)	Female (*n*=109)
Age (year)	71.9±7.4	74.2±7.0	70.9±7.4
Heigth (cm)	155.6±8.8	163.5±7.3	152.2±7.1
Weight (kg)	56.6±10.2	62.8±10.3	53.9±9.0
BMI (kg/m^2^)	23.3±3.1	23.4±2.9	23.2±3.1
GS (kg)	21.8±6.9	29.0±5.8	18.5±4.4
5 TCST (s)	10.7±3.1	10.2±3.0	10.9±3.1
5 MI (kg/m^2^)	6.1±1.0	7.0±0.8	5.7±0.8

BMI: body mass index; GS: grip strength; 5 TCST, times chair stands test; SMI: skeletal mascle index: SD, standard deviation

### Sarcopenia screening

The diagnostic criteria of AWGS 2019 were applied to screen eligible participants [9]. Briefly, the cutoffs for low skeletal muscle mass are as follows: < 7.0 kg/m^2^ in men and < 5.7 kg/m^2^ in women by BIA measurement (InBody 770, Seoul, Korea). The cutoffs for low muscle strength are as follows: < 28 kg in men and < 18 kg in women by handgrip strength test using a standard electronic handgrip dynamometer (CAMRY, Guangzhou, China). Two of the four communities had limited space for testing, and to ensure consistency across the experimental protocol, the 5-times chair sit-to-stand test was chosen to evaluate physical performance, instead of tests that require more space. A cutoff of ≥ 12 s was considered to indicate low physical performance. A participant would be considered to be sarcopenic when he or she has low muscle mass, and one or both the other two conditions: low muscle strength, and low physical performance. In our study, participants were included in the healthy group when their muscle mass, muscle strength, and physical performance were all within the normal range.

### Experimental protocol

**Fig. 1:**
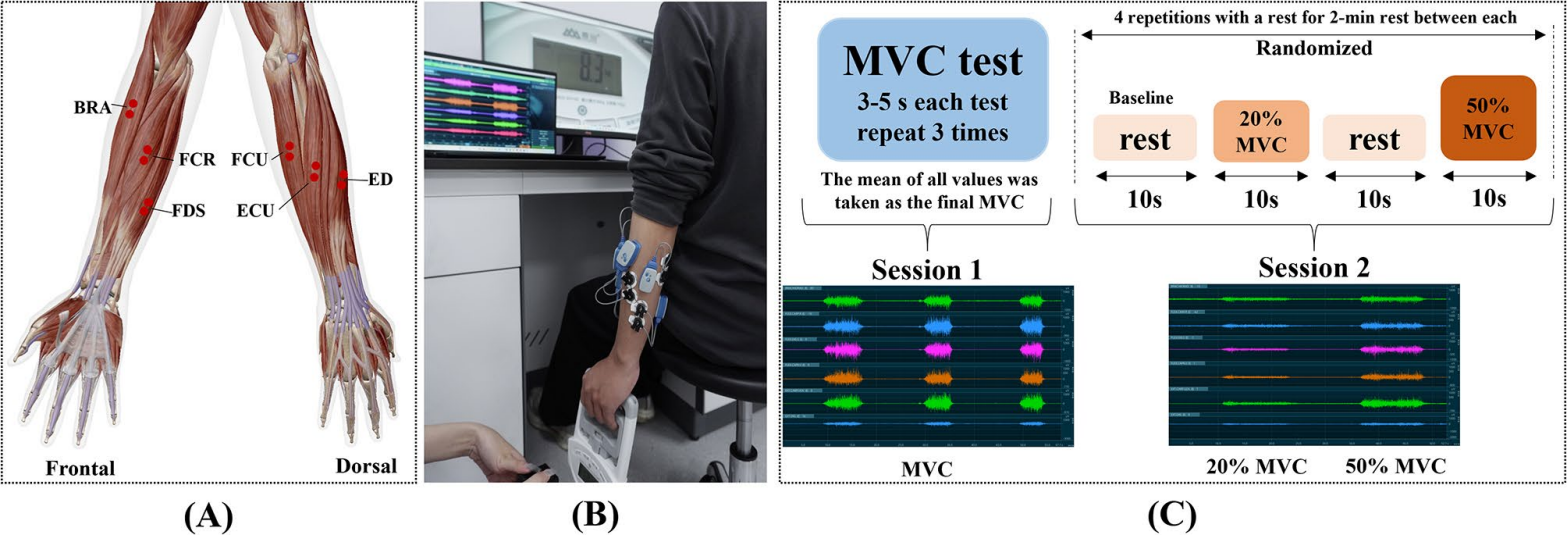
Experimental Protocol. (**A**) The location of the electrodes. (**B**) Experimental process. (**C**) Experimental protocols. MVC, maximal voluntary contraction; BRA, brachioradialis; FCR, flexor carpi radialis; FDS, flexor digitorum superficialis; FCU, flexor carpi ulnaris; ECU, extensor carpi ulnaris; ED, extensor digitorum

At the beginning of the experiment, the forearm skin of the participant was shaved lightly and wiped with an alcohol pad to provide a good condition of sEMG signal acquisition. Six Ag/AgCl electrode pairs (Kendall H124SG, CardinalHealth Inc., Dublin, Ohio, USA) were attached to the muscle abdomen of the brachioradialis (BRA), flexor carpi radialis (FCR), flexor digitorum superficialis (FDS), flexor carpi ulnaris (FCU), extensor carpi ulnaris (ECU), and extensor digitorum (ED), see Fig. 1A. The distance between the electrodes was 2 cm. A wireless sEMG system (Ultium EMG, Noraxon Inc., Scottsdale, USA) was used to record the sEMG signals at a sampling rate of 2000 Hz and a gain of 1000.

During the experiment, the participants were seated comfortably on a chair facing a computer screen to receive visual feedback. The experiment was divided into two sessions (Fig. 1B **and** Fig. 1C). **In session 1**, the participants were instructed to place their arms at the sides of their bodies naturally and perform maximal voluntary contraction (MVC) by gripping the hand-held dynamometer. The maximum grip force that the participant could maintain steadily for 3–5 s was recorded. And each participant would repeat MVC contractions three times, with sufficient resting between them. The mean force value out of the three MVC contractions was considered to be the MVC force value of the participant. After a resting period, **session 2** of the experiment would begin. The participants were asked to perform a series of sub-maximal contractions and track the target force which was displayed on the computer screen. At the beginning of each sub-maximal contraction, there is a resting period of 10 s, with a digital count-down displayed on the computer screen. Data in this resting period served as the baseline data with which data from subsequent ‘active’ periods would be referenced. A number, either 20% MVC or 50% MVC would be displayed next to the count-down, indicating the target force level to be reached in the coming sub-maximal contraction. When the count-down expired, the participant would then compress the dynamometer, reach either 20% MVC force and 50% MVC force, and maintain the force level for 10 s. Each participant would perform four sub-maximal contractions at the two levels in random order. And additional rest periods between contractions would be provided upon requests from the participants to avoid short-term fatigue. An entire experiment would last no longer than 30 min, a duration well-tolerated by all participants.

### Data processing and feature extraction

An experienced surface EMG expert inspected the acquired data to determine if there were abnormal channels with excessive noise and data from two participants were discarded. The acquired sEMG signals were digitally filtered by notch filters of integral multiples of 50 Hz and bandpass 3rd order Butterworth filter between 10 Hz and 500 Hz. A three-second segment from each sub-maximal contraction was selected for subsequent analysis by visual inspection for stationarity. Then, 200-ms data windows were extracted with an increment step of 50 ms. A total of nine representative features were extracted from the data windows. Six of which were the typical Hudgins time-domain features, including root mean square (RMS), mean absolute value (MAV), integrated EMG (iEMG), waveform length (WL), zero crossing (ZC), and slope sign change (SSC) [42]. These features are widely used in sEMG signal analysis due to their computational simplicity and effectiveness in extracting time-domain information. In addition, to encode more comprehensive time and frequency domain information at the same time, three time-frequency features were also extracted from continuous wavelet transform (CWT) coefficients, namely the absolute power (CWT_power), kurtosis (CWT_kurtosis), and wavelet entropy (WE) [43]. First, CWT coefficients ${C}_{a,b}$were computed according to Eq. (1) at each scale *a* and time step *b* [44]:

1 $${C}_{a,b} = CWT(a,b) = \frac{1}{\sqrt{a}}{\int }_{-\infty }^{+\infty }x\left(t\right)\psi *\left(\frac{t-b}{a}\right) \text{d}t (a>0, b\in \mathbb{R})$$

where $x\left(t\right)$ is the EMG signal, a function of time $t$, $\psi \left(t\right)$ represents the chosen mother wavelet function, and the asterisk $*$ indicates the complex conjugate. In this study, the Morlet wavelet described in Eq. (2) is chosen as the mother wavelet function for its computational simplicity and effectiveness in EMG and EEG signal processing [44]:

2 $$\psi \left(t\right) = {e}^{\text{i}{\omega }_{0}t}{e}^{-{t}^{2}2}$$

where ${\omega }_{0}$ is the mother wavelet center frequency, and i indicates the imaginary part. To capture the distribution features of the computed CWT coefficients, at each scale *a*, power is calculated using Eq. (3), then averaged over all scales.:

3 $${\text{C}\text{W}\text{T}\_\text{p}\text{o}\text{w}\text{e}\text{r}}_{\text{a}} = \frac{1}{n}\sum _{b=1}^{n}{\left|{C}_{a,b}\right|}^{2}$$

where *n* is the total number of sampling points. The scale factor *a* for this study is chosen as the set: $\left\{3.6, 4.6, 5.6, \ldots , 62.6\right\}$to represent effective frequency ranges (10–500 Hz) in our study [44]. Using the calculated CWT_power, wavelet entropy at each scale is computed according to Eq. (4):

4 $$\text{W}\text{E}= -\sum _{a}{h}_{a} log\left({h}_{a}\right), {h}_{a}=\frac{{\text{C}\text{W}\text{T}\_\text{p}\text{o}\text{w}\text{e}\text{r}}_{\text{a}}}{\sum _{a}{\text{C}\text{W}\text{T}\_\text{p}\text{o}\text{w}\text{e}\text{r}}_{\text{a}}}$$

and then averaged over all scales. Besides the above commonly used CWT features, since the MU activation is closely related to the probability distribution of sEMG signals [45], we further calculated the kurtosis of the probability density function (PDF) of CWT coefficients. First, empirical PDF is estimated using the KernelDensity function in the scikit-learn package in Python, and then the kurtosis of the PDF is computed according to Eq. (5):

5 $$\eqalign{ CW{T_k}urtosis\; = & \;\left[ {{{m(m + 1)} \over {(m - 1)(m - 2)(m - 3)}}\sum\limits_{i = 1}^m {{{\left( {{{{X_i} - \bar X} \over S}} \right)}^4}} } \right]\; \cr & - {{3{{(m - 1)}^2}} \over {(m - 2)(m - 3)}} \cr}$$

where *m* is the total number of discrete points used to estimate the PDF (which is 100 in typical settings), ${X}_{i}$ represents the *i*-th PDF value, $\stackrel{-}{X}$ indicates the sample average of PDF values, and *S* is the sample standard deviation of all *X*.

After the initial feature extraction, all features were channel-specifically normalized using MVC-normalization [45]. Specifically, the sEMG features extracted from each channels at each contraction level (20% MVC and 50% MVC) were normalized using the average feature values obtained from three MVC tests of the same channel in **session 1**.

### Statistical analysis

Since unequal variances and deviations from the Gaussian distribution were observed in most of the features between two groups based on Levene’s Test for unequal variances and Kolmogorov-Smirnov Test for goodness-of-fit, parametric tests such as the t-test were not applicable. Hence, non-parametric tests were utilized in this study. Firstly, to compare the differences in sEMG signal characteristics between the sarcopenic group and the healthy group, the average of each feature was computed across all six channels and all four trials. Then, statistical analyses were performed employing the Mann-Whitney U Test with a significance level of 0.05. Additionally, to compare the differences in sEMG signal characteristics at different contraction levels within each group, statistical analyses were performed employing the Wilcoxon Matched Pairs Signed Rank Test with a significance level of 0.05. All analyses were performed with SPSS version 25.0 (SPSS Inc., United States).

### Classification using machine learning model

To efficiently identify sarcopenic patients using sEMG signals, we implemented a voting classification model with binary labels: sarcopenic versus healthy [40]. The voting classifier is an ensemble ML model that makes inferences based on majority voting of several chosen ML methods [46]. The advantage of a voting classifier is to avoid potential limitations and biases of any single ML model by combining the results of several ML models through a voting strategy [46]. In this study, a linear kernel support vector machine (SVM) [47], a random forest (RF) [48], and a gradient boosting machine (GBM) [49] were chosen to ensemble the voting classifier. SVM is a classic ML method that classifies data by finding the best hyperplane in the feature space that divides the groups; it is friendly to small-sized and linearly-dividable samples [40]. RF is a tree-based method employing the bagging technique; it is also stable against small samples [48]. In contrast, GBM is an ensemble learning method that employs the gradient boosting technique; it typically performs better on large enough datasets with proper settings of hyperparameters such as the learning rate [49]. To combine the merits of these three different ML models, a weighted soft voting ensemble (SVE) scheme [46] according to Eq. (6) was implemented to predict the output label $\widehat{y}$ using the above three classifiers, in which the voting weight ${w}_{i}$for the *i*-th classifier was determined by the Grid-Search method to achieve the highest sensitivity in the training process:

6 $$\widehat{y} = \text{a}\text{r}\text{g} \underset{j}{\text{max}}\sum _{i=1}^{3}{{w}_{i}P}_{i,j} \text{w}\text{h}\text{e}\text{r}\text{e} j=\left\{\text{0,1}\right\}$$

here, ${P}_{i,j}$ is the predicted probability of the *j*-th class using the *i*-th classifier. In this task, $j=0$ represents the healthy group, while $j=1$ represents the sarcopenia group. Training and testing were done using the five-fold cross validation (CV) method on 93 subjects, introduced in the next section.

### Evaluation and model interpretability

The performance of the voting classifier is evaluated using a subject-level five-fold cross-validation scheme to assess the stability of the classification model across the participant pool [50]. For computational efficiency and stability purposes, a Shuffle Split was first employed to divide 93 participants into five folds; within each fold, the number of sarcopenic participants and the number of healthy participants only differed by one or less. Then a leave-one-out CV method was performed on each fold. Evaluation metrics include accuracy, sensitivity, specificity, F1-score, and the area under the receiver operating characteristic (ROC) curve (AUC) [51]. Formulae of these indices can be found in references [51], and the ROC curve is obtained by pairs of the false positive rate versus the true positive rate for different levels of discretized decision thresholds, which were often set as the set of distinct values of predicted probability scores using the model, in the range between zero and one [51]. Additionally, to evaluate the advantages of the voting classifier on performance, each single classifier that ensembles the voting was trained and tested alone, and then we compared the results with our voting classifier. In addition to accuracy performance, model interpretability and the importance of features are essential for clinical applications to interpret the classification model, making it more understandable. Recently, the SHapley Additive exPlanations (SHAP) method for model interpretation was proposed by Su-In Lee et al. [52], using the Shapley value to estimate the impact score of each feature on the classification outcomes. By using SHAP method, the internal classification process is taken out of the ‘black box’, and the contribution of discriminative information of different features is analyzed and interpreted. All the analysis in this study was implemented using Python version 3.11.3, Scikit-learn version 1.3.0, PyWavelets version 1.4.1, and Shap version 0.42.1.

## Results

### Participants characteristics

After the data quality check, data from two participants were excluded (one in each group). Subsequently, participants in the non-sarcopenic group with normal values for muscle mass, muscle strength, and physical performance were assigned to a healthy group (*n* = 45). For some elderly partcipants (*n* = 64) who meet only one of the AWGS diagnostic criteria, although we cannot diagnose them with sarcopenia, they cannot be classified as healthy individuals either, as they have functional impairments in terms of muscle strength or muscle mass. Data from these 64 participants was not further analyzed in the current study. See Table 2 for further details.

**Table 2:** Descriptive statistics of the sarcopenia group and the healthy group represented as mean (±SD)

	Healthy group (*n*=45)			Sarcopenia group (*n*=48)		
	Total (*n*=45)	Male (*n*=16)	Female (*n*=29)	Total (*n*=48)	Male (*n*=17)	Female (*n*=31)
Age (year)	68.6±5.8	69.7±5.6	67.7±5.9	75.3±7.7	77.9±6.6	73.8±8.0
Heigth (cm)	160.3±8.5	168.5±7.4	155.8±4.9	151.8±9.0	59.5±6.1	147.4±7.1
Weight (kg)	63.7±10.3	70.7±12.1	59.8±6.7	51.7±8.3	57.9±5.0	48.4±7.9
BMI (kg/m^2^)	24.7±2.9	24.8±2.9	24.7±2.9	22.4±2.5	22.6±1.8	22.2±2.9
GS (kg)	26.6±6.9	34.1±5.4	22.4±3.0	17.7±5.2	23.8±2.2	14.4±2.8
5 TCST (s)	9.9±2.0	10.3±2.7	9.7±1.5	10.6±3.2	10.1±3.3	10.9±3.2
5 MI (kg/m^2^)	6.9±0.9	7.7±0.7	6.4±0.6	5.6±0.8	6.5±0.3	5.1±0.4

BMI: body mass index; GS: grip strength; 5 TCST, times chair stands test; SMI: skeletal mascle index: SD, standard deviation

### sEMG features analysis

**Fig. 2:**
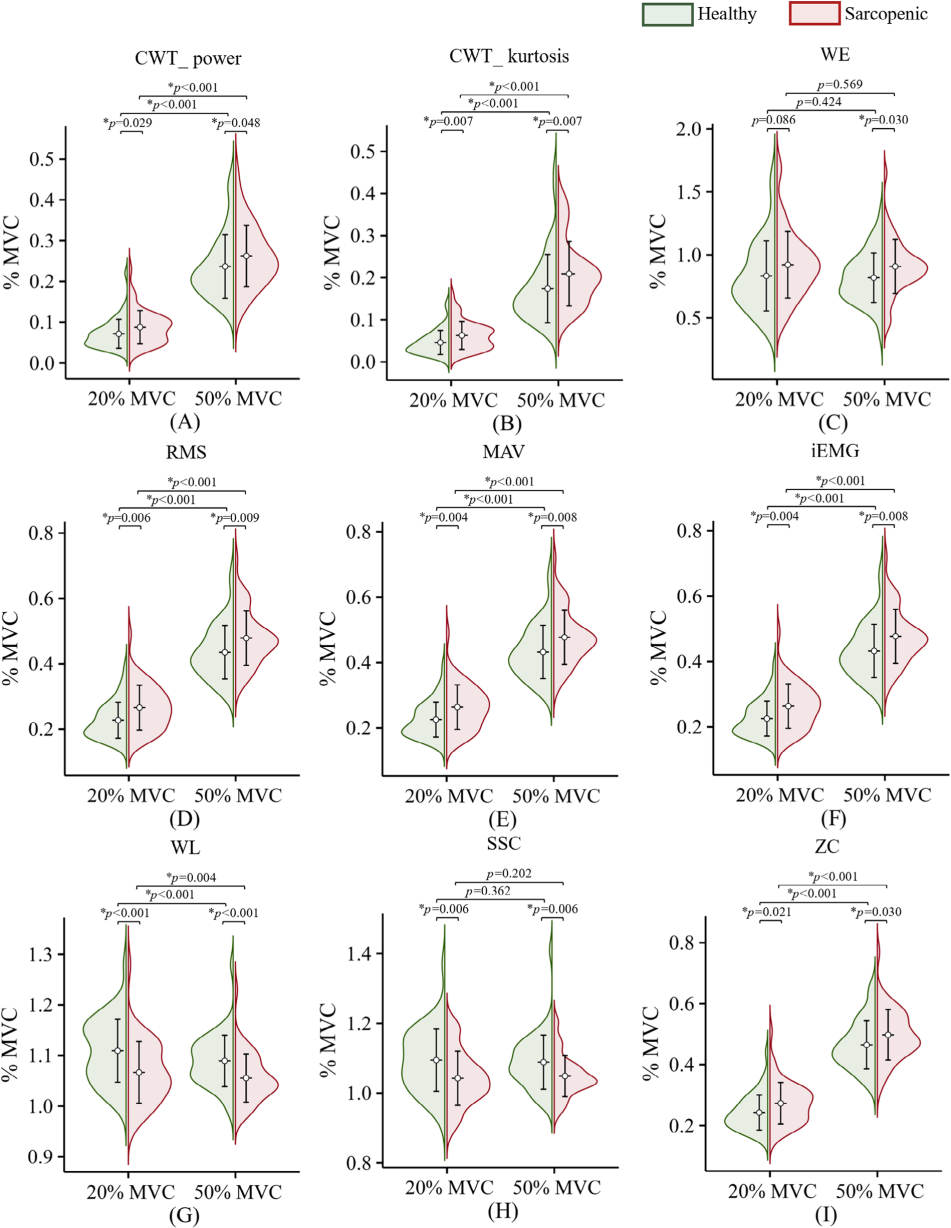
The violin plot depicting all sEMG features between the sarcopenia group and the healthy group in both MVC conditions. CWT_power (continuous wavelet transform coefficients power), WE (wavelet entropy) CWT_kurtosis (continuous wavelet transform coefficients kurtosis), RMS (root mean square), MAV (mean absolute value), iEMG (integrated electromyography), WL (waveform length), SSC (slope sign change), ZC (zero crossing), * *p* < 0.05. The mean and standard deviation of each feature are shown through the circle makers and error bars, respectively

The violin plot (Fig. 2) was applied to represent all sEMG features between the sarcopenic group and the healthy group in both MVC conditions. The Levene?s test confirmed the non- homogeneity of the variance (all the *p*-values were > 0.05), and the Mann-Whitney U test revealed that statistically significant differences were found between the sarcopenic group and the healthy group in all sEMG features in both MVC conditions with one exception: the WE in 20% MVC level (Fig. 2C). Meanwhile, the Wilcoxon Matched Paired Signed Rank test revealed that statistically significant differences were found within the sarcopenic group and the healthy group in all sEMG features between 20% MVC and 50% MVC with exceptions: WE (Fig. 2C) and SSC (Fig. 2H). Interestingly, WL behaved differently from other features that reflect EMG energy and complexity, including CWT_power (Fig. 2A), RMS (Fig. 2D), MAV (Fig. 2E), and iEMG (Fig. 2F). The average WL value was higher in the healthy group than in the sarcopenic group in both conditions and was also higher in the 20% MVC than in the 50% MVC within both groups (Fig. 2G). But obviously, the sarcopenic group showed a tendency of higher complexity in sEMG signals during muscle contraction, and this trend increased with the contraction levels.

### Machine learning results

**Fig. 3:**
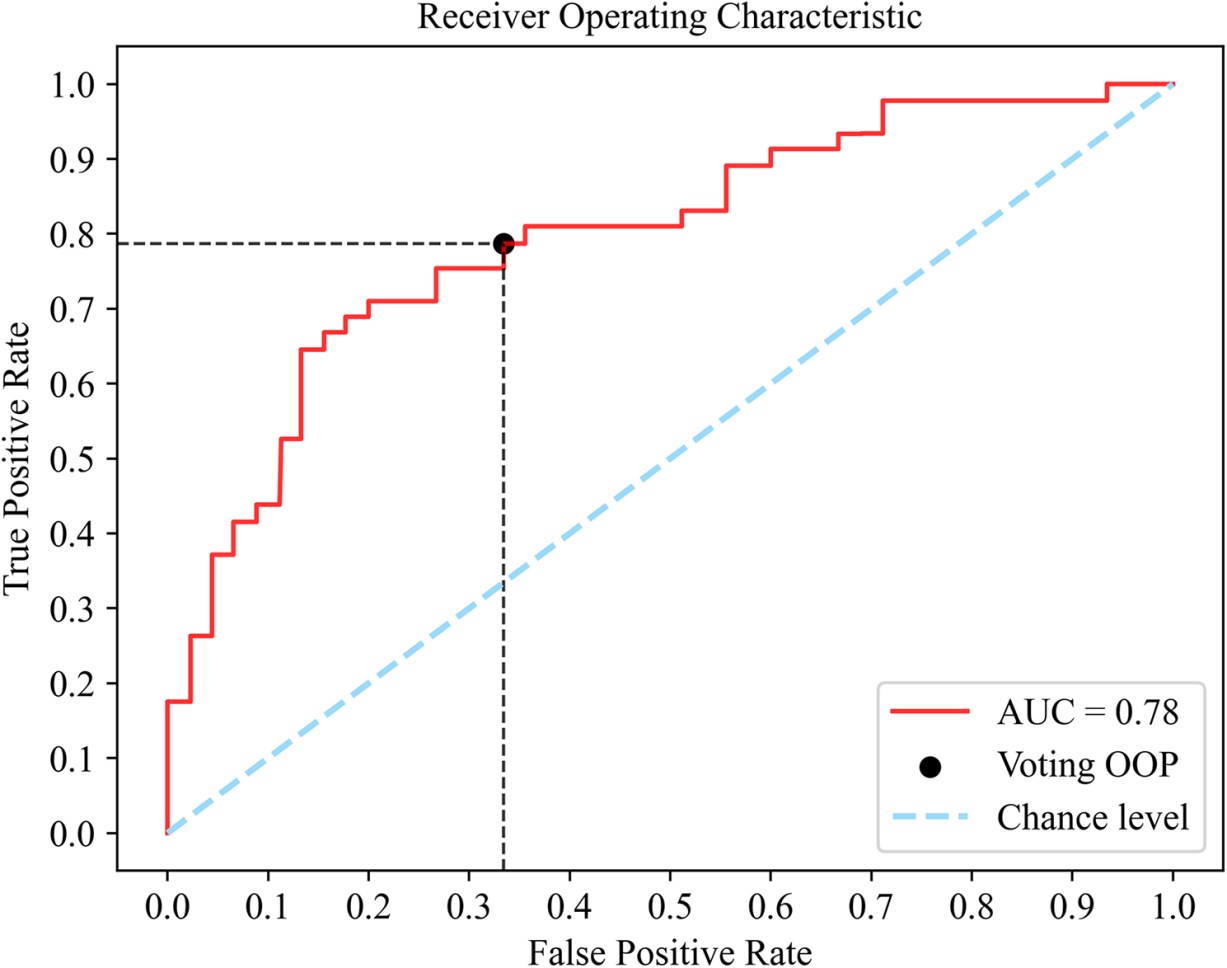
ROC-AUC result of the voting classifier. The blue dotted line indicates the chance level (i.e., random guessing), and the red solid line represents the ROC result curve of the model. The black point indicates the optimal operating point (OOP) for the model

Channel selection and feature selection were first employed to improve the model performance. A combination of channels two and three was selected while dropping the three least important features that had the lowest SHAP values. Then, Grid-Search was implemented to determine optimal hyperparameters and voting weights. Using the five-fold CV described in Sect. 2.7 across all 93 participants, the performances of SVM, RF, GBM, and the voting classifier are summarized in Table 3 in the form of mean ($\pm$ standard deviation). In general, the voting classifier achieves the highest accuracy and sensitivity among all the models included. The accuracy and sensitivity were 0.73 ± 0.07 and 0.79 ± 0.07, respectively. The ROC curve result of the voting classifier is presented in Fig. 3 with an AUC score reaching 0.78. In the ROC curve, the more the curve approaching the upper-left corner, making the area under the curve larger, the more accurate our model can predict. Since the primary goal is to develop an early screening tool, high sensitivity should be prioritized when considering the balance between sensitivity and specificity. As such, the optimal operating point (OOP) of ROC was chosen by the rule that it has the highest sensitivity while specificity is above the 0.65 threshold [39].

**Table 3:** Classification results with five-fold CV represented as mean (±SD)

	Accuracy	Sensitivity	Specificity	FI-score
SVM	0.67 (±0.04)	0.69 (±0.04)	0.64 (±0.03)	0.67 (±0.02)
RF	0.70 (±0.06)	0.73 (±0.06)	0.64 (±0.03)	0.70 (±0.04)
GBM	0.70 (±0.06)	0.75 (±0.08)	0.62 (±0.04)	0.70 (±0.06)
Voting	0.73 (±0.07)	0.79 (±0.07)	0.67 (±0.07)	0.73 (±0.06)

SVM, support vector machine; RF random forest; GBM, gradient boosting machine; CV, cross validation; SD, standard deviation

### Model interpretability

**Fig. 4:**
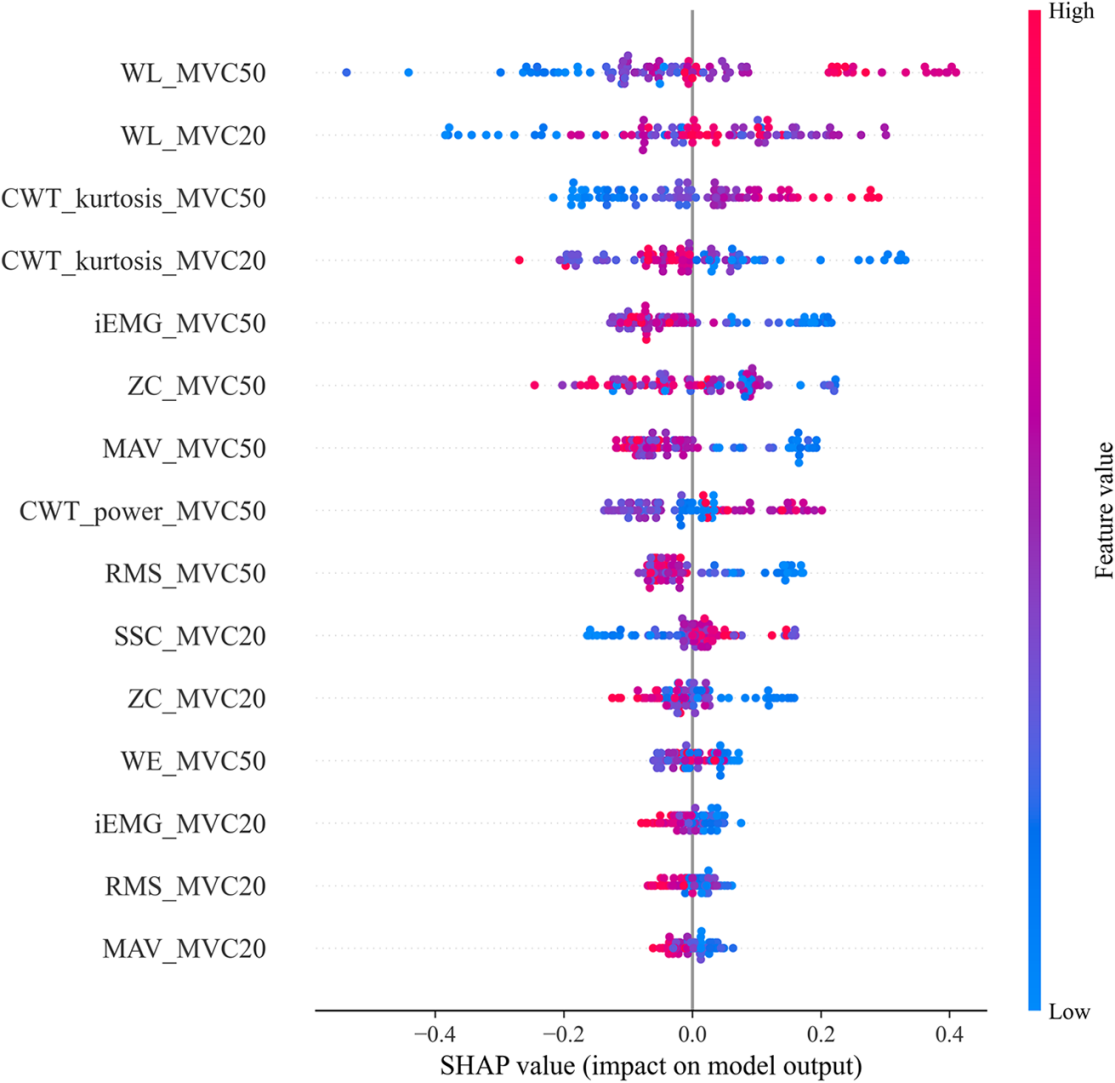
SHAP summary plot. Each point represents a SHAP value for a feature and a subject. Vertical axis is the features and horizontal axis is the SHAP values. Redder color indicates higher feature values

Feature impact scores of the voting classifier estimated by the SHAP method using all 93 participants are summarized in (Fig. 4). In the SHAP summary plot, each point represents a computed SHAP value for one feature and one subject. Features were ordered by their importance score from top to bottom along the vertical axis. Feature values were denoted by the color, with higher feature values being deeper shades of red while lower feature values being deeper shades of blue. As mentioned in the previous section, three features that had the lowest SHAP scores were removed to improve the model performance: CWT_power at 20% MVC, WE at 20% MVC, and SSC at 50% MVC. As shown in the graph, WL and CWT_kurtosis at both MVC levels acquired the highest impact score with an obvious leading gap for predicting the outcome using our Voting classifier. Notice that WL also had the lowest p-value between the groups in our statistical analysis. The SHAP result aligns with recent studies on how the motor unit activation of abnormal elders can differ from that of healthy populations, and the difference could be visualized in the core shape of the probability density function (PDF) of sEMG signals [45]. The interpretation of this result relating to motor units is further discussed in the Discussion section below.

## Discussion

This study aimed to investigate the possibility of using sEMG as an early screening tool for sarcopenia in community-dwelling settings. A set of features of sEMG have been investigated, and significant differences between the two groups of community-dwelling older adults were identified using some of these features. Hence, it can be concluded that these features have the potential to capture sarcopenia-related differences. Furthermore, a voting classifier ML model was implemented with these features to perform the sarcopenia screening task. Moderate results were presented in identifying sarcopenic patients through these features, including a high sensitivity and acceptable accuracy, which are desirable properties for early-screening applications. Lastly, the SHAP method was performed to estimate features’ impacts on predicting classification outcomes.

The current studies on using sEMG for diagnosing sarcopenia almost focused on lower limb and trunk muscles. Habenicht et al. [53] found that the instantaneous median frequency (IMDF) values of back muscles changed more rapidly with time in younger than in older individuals. A smaller percentage of fast fatiguing MUs had resulted in the less pronounced IMDF slope change in older individuals. Tian et al. [54] observed that the RMS of sEMG in lower extremity muscles was significantly higher in the young compared to the elderly only at 75% of MVC, while there was no significant difference at 25% and 50% MVC. Our study found that no matter in 20% MVC or 50% MVC, the time-domain features of forearm muscles in the sarcopenic group were significantly different from those of the healthy elderly group. To the best of our knowledge, this is the first study to investigate sarcopenia through sEMG in forearm muscles. It is worth mentioning that during the screening process for sarcopenia, we noticed that the decline in upper limb function (i.e., GS) in elderly individuals was more significant than the decline in lower limb function (i.e., 5 TCST). This may suggest that the sEMG signals of upper limbs are more sensitive to sarcopenia compared to lower limbs.

With typical adult aging, the number of functional MUs is diminished and is likely coupled with incomplete compensatory reinnervation of muscle fibers [55]. Thus, neuromuscular deficits with aging may be related in part to the loss of MUs but also to deficits in neuromuscular transmission and MU stability secondary to the denervation process [56]. Our findings revealed that the sarcopenia group exhibited greater signal complexity compared to the healthy elderly group. The higher signal complexity may be attributed to altered MU properties, resulting in decreased stability of NMJ propagation and increasing variation in MUAP size and shape. And further may indicate that MUs during sarcopenia have more muscle fibers, leading to a higher proportion of innervation. These findings are consistent with the previously described pathological mechanism of sarcopenia [25]. However, to provide solid evidence of morphological changes in sarcopenia, either invasive EMG techniques (such as needle or fine-wire EMG) or high-density surface EMG (HD-sEMG) techniques need to be employed. Imrani et al. [57] demonstrated that analysis of HD-sEMG data from the rectus femoris during sit-to-stand trials enables the assessment of muscle aging. Considering the frail conditions of sarcopenia patients, invasive EMG is not practical. Additionally, the current HD-sEMG electrodes’ conformability and skin preparation requirements make their use challenging. Therefore, it is imperative to explore novel HD-sEMG electrode materials with enhanced conformability and simplified deployment procedures [58–60].

Regarding the interpretable ML results, WL and CWT_kurtosis turned out to be the most impactful ones in our voting classification model among all features. The WL mainly reflects amplitude information of the signal while the CWT_kurtosis represents the deviation from Gaussian distribution after wavelet transformation [43]. As in other studies that used ML methods for sarcopenia screening, amplitude features tend to perform well in models like SVM and tree-based models [38, 39]. Additionally, the voting classifier combined these merits and even performed better than each of the single classifiers regarding sensitivity scores. Besides traditional time-domain features, differences observed in the CWT_kurtosis could be caused by the relation of how MU activation patterns affect the probability distribution of sEMG signals [45]. As discussed above, sarcopenic participants tend to have distinct MU properties during contractions compared to healthy participants, and thus these property differences could be reflected in the shape of PDF of sEMG signals. Our results indeed indicated that sarcopenic participants tend to have higher kurtosis values, meaning that the PDF deviates further from the Gaussian distribution and exhibits a higher level of signal complexity.

However, we acknowledge that some limitations of this study still exist. Firstly, according to the diagnostic criteria of AWGS, sarcopenia and severe sarcopenia should be distinguished, we classified both as sarcopenia, so only a binary classification was implemented. Another limitation was the simplified protocol. To perform sarcopenia early screening in community circumstances, our experimental protocol was designed to be relatively simple, only including upper-limb contraction data. While this embraced the benefits of convenient and speedy tests in communities, sEMG signals detected through this protocol could suffer from homogeneity and low quality, making the ML classification task more difficult. Secondly, due to strictly screening community-dwelling older adults for sarcopenia according to the AGWS diagnostic criteria, there was indeed a significant age difference between the healthy group and the sarcopenic group. However, the clear age differences between the two groups were due to the nature of the disease rather than any methodological age-bias. The EMG-based approach we proposed, along with the AWGS, is merely used to identify differences in muscle mass and muscle function, both of which are age-agnostic in nature. Thirdly, for better interpretability purposes, complicated deep learning methods that can potentially achieve higher classification accuracy through learning the features themselves were not implemented in this study [38, 39]. Although deep learning methods exhibit generally better performances in classification, the data size and quality requirements exceed those of the currently collected data in communities. Plus, deep learning methods will sacrifice interpretability since the features are to be learned and extracted themselves, providing much less insight into the clinical importance of certain features. Instead, we manually extract time-frequency domain features and investigate their importance in our classification task. Compared to deep learning models for sarcopenia classification, our methodology would have lower accuracy performance in general [61]. To improve, more potentially sensitive features such as complexity, orderliness, and core shape modeling could be further investigated and utilized in ML. Besides, data augmentation is a great tool to enhance and expand the dataset to fit into deep learning methods. Despite of lack of interpretability, data augmentation combined with deep learning models might be beneficial in achieving better accuracy. Many of the aforementioned limitations regarding extracting sarcopenic information from EMG stem from the cross-sectional data acquisition approach. If EMG data could be collected over a longer time span and outside laboratory conditions, the full potential of EMG could be realized. This would necessitate the use of flexible sensing technologies, such as comfortable wearable electronics and mobile health devices, among others [62, 63].

## Conclusions

In the practical application of sarcopenia screening, there is a need for faster, time-saving, and community-friendly detection methods. This study proposed a method for community-based sarcopenia screening based on sEMG signals of forearm muscles. The dataset consists of 45 healthy elderly individuals and 48 elderly individuals with sarcopenia. Using a voting classification ML model, the accuracy exceeds 70% and the sensitivity exceeds 75%, indicating moderate classification performance. Interpretable results obtained from the SHAP model suggest that MU activation mode may be a key factor affecting sarcopenia.

## Data Availability

Anonymized data are made available upon request.

## Abbreviations

AWGS 2019: The Asian Working Group for Sarcopenia in 2019
EWGSOP: The European Working Group on Sarcopenia in Older People
BIA: Bioelectric impedance
DXA: Dual-energy X-ray absorptiometry
CT: Computed tomography
High-density surface EMG: HD-sEMG
MRI: Magnetic resonance imaging
MU: Motor unit
NMJ: Neuromuscular junction
MUAPs: Motor nunits action potentials
EMG: Electromyography
ML: Machine learning
MVC: Maximal voluntary contraction
RMS: Root mean square
MAV: Mean absolute value
iEMG: Integrated electromyography
WL: Waveform length
ZC: Zero crossing
SSC: Slope sign change
CWT: Continuous wavelet transform
CWT_power: Absolute power of continuous wavelet transform
CWT_kurtosis: The kurtosis of continuous wavelet transform
WE: Wavelet entropy
SVM: Support vector machine
RF: A random forest
GBM: Gradient boosting machine
SVE: Soft voting ensemble
CV: Cross validation
SHAP: SHapley Additive exPlanations
ROC: The receiver operating characteristic curve
AUC: Area under the curve
PDF: Probability density function
OOP: The optimal operating point
IMDF: Instantaneous median frequency
